# Environmental and Occupational Risk Factors of Amyotrophic Lateral Sclerosis: A Population-Based Case-Control Study

**DOI:** 10.3390/ijerph17082882

**Published:** 2020-04-22

**Authors:** Tommaso Filippini, Marina Tesauro, Maria Fiore, Carlotta Malagoli, Michela Consonni, Federica Violi, Laura Iacuzio, Elisa Arcolin, Gea Oliveri Conti, Antonio Cristaldi, Pietro Zuccarello, Elisabetta Zucchi, Letizia Mazzini, Fabrizio Pisano, Ileana Gagliardi, Francesco Patti, Jessica Mandrioli, Margherita Ferrante, Marco Vinceti

**Affiliations:** 1 CREAGEN-Environmental, Genetic and Nutritional Epidemiology Research Center, Department of Biomedical, Metabolic and Neural Sciences, University of Modena and Reggio Emilia, 41125 Modena, Italy; carlotta.malagoli@unimore.it (C.M.); federica.violi@unimore.it (F.V.); l.iacuzio@ausl.mo.it (L.I.); elisa.arcolin@gmail.com (E.A.); marco.vinceti@unimore.it (M.V.); 2 Department of Biomedical, Surgical and Dental Sciences, University of Milan, 20122 Milan, Italy; marina.tesauro@unimi.it (M.T.); michela.consonni@unimi.it (M.C.); 3 Department of Medical, Surgical Sciences and Advanced Technologies “G. F. Ingrassia”, Catania University, 95123 Catania, Italy; mfiore@unict.it (M.F.); olivericonti@unict.it (G.O.C.); antonio.cristaldi81@gmail.com (A.C.); pietro.zuccarello@unict.it (P.Z.); patti@unict.it (F.P.); marfer@unict.it (M.F.); 4 Azienda USL-IRCCS di Reggio Emilia, 42122 Reggio Emilia, Italy; 5 Department of Public Health, Local Health Unit, 41121 Modena, Italy; 6 Neurology Unit, Department of Biomedical, Metabolic and Neural Sciences, University of Modena and Reggio Emilia, 41125 Modena, Italy; elibettizucchi@gmail.com; 7 ALS Centre Department of Neurology, ‘Maggiore della Carità’ University Hospital, 28100 Novara, Italy; letizia.mazzini@uniupo.it (L.M.); ileanagagliardi91@gmail.com (I.G.); 8 Neurological Rehabilitation Division, Policlinico San Marco di Zingonia, 24046 Zingonia (BG), Italy; fabrizio.pisano@grupposandonato.it; 9 Neurology Unit, Department of Neuroscience, S. Agostino Estense Hospital, Azienda Ospedaliero Universitaria di Modena, 41126 Modena, Italy; mandrioli.jessica@aou.mo.it; 10 Department of Epidemiology, Boston University School of Public Health, Boston, MA 02118, USA

**Keywords:** amyotrophic lateral sclerosis, occupational factors, environmental factors, case-control study

## Abstract

*Objectives:* Amyotrophic lateral sclerosis (ALS) is a progressive and fatal neurodegenerative disease with still unknown etiology. We aimed at investigating the association between environmental and occupational factors with ALS risk. *Methods:* We performed a population-based case-control study in four Italian provinces (Catania, Modena, Novara, and Reggio Emilia) by administration of tailored questionnaires to ALS cases (*n* = 95) and randomly selected population referents (*n* = 135). We estimated ALS risk by calculating the odds ratio (OR) with its 95% confidence interval (CI) using an unconditional logistic regression model. *Results:* We found a positive association with disease risk for history of occupation in the agricultural sector (OR = 2.09, 95% CI 0.79–7.54), especially for longer than 10 years (OR = 2.72, 95% 1.02–7.20). Overall occupational exposure to solvents also suggested a positive association, especially for thinners (OR = 2.27, 95% CI 1.14–4.54) and paint removers (OR = 2.01, 95% CI 0.90–4.48). Both occupational and environmental exposure to electromagnetic fields show a slightly increased risk with OR = 1.69 (95% CI 0.70–4.09) and 2.41 (95% CI 1.13–5.12), respectively. Occupational but not environmental exposure to pesticides (OR = 1.22, 95% CI 0.63–2.37), particularly fungicides, and exposure to metals (OR = 4.20, 95% CI 1.88–9.38), particularly lead, mercury, and selenium, showed an imprecise but positive association. Finally, there was an indication of increased risk for living in proximity to water bodies. *Conclusions:* Despite the caution that needs to be used due to some study limitations, such as the low number of exposed subjects and the possibility of recall bias, these results suggest the potential role of some environmental and occupational factors in ALS etiology.

## 1. Introduction

Amyotrophic lateral sclerosis (ALS) is a rare but fatal degenerative disease of the central nervous system. The epidemiology of ALS has been widely studied in several countries, including European ones [1,2,3,4,5]. The incidence is approximately 1–3 cases per 100,000 and it has been increasing in recent decades [6]. ALS is classified as either sporadic or familial, accounting for approximately 90% and 10%, respectively. Major advances have been made in the understanding of the genetic causes of the disease, whereas the contribution of environmental factors in both sporadic and familial ALS has been more difficult to assess, and large-scale studies have not yet revealed replicable and definitive evidence for them [7]. Several environmental and occupational factors have been investigated [8,9,10,11], although the evidences are still inconsistent. In particular, smoking habits, military service, exposure to electromagnetic fields, cyanotoxins, and chemical agents such as pesticides and solvents have been addressed [9,12,13]. An association between the disease and exposure to heavy metals and other trace elements including lead, particularly from occupational activities, seems to be supported by strong epidemiological evidence [14,15]. Similarly, environmental exposure to the metalloid selenium in its inorganic form has been linked to increased ASL incidence [16,17]. In addition, a higher frequency of ALS has been reported among athletes compared to the general population [11], suggesting that trauma associated to physical activity may increase disease risk. However, several limitations hampered the identification of factors with a high certainty of evidence [14]. These include, for example, the limited number and size of the studies that investigated these environmental risk factors, the difficulties of conducting research on a rare neurological disease, the high cost, and the limited number of population-based investigations. In this study, we sought to investigate environmental and occupational factors potentially associated with the ALS risk in an Italian population, using a populations-based case-control study design.

## 2. Methods

We designed a population-based case-control study to investigate the role of environmental risk factors in ALS etiology. Study methods have been already described in detail [18]. We recruited the ALS cases diagnosed in the period 2008–2011 in Catania, Modena, and Reggio Emilia provinces, and in the period 2002–2012 in Novara province.

We identified eligible ALS cases through multiple population-based sources. These sources were two ALS Registries, namely the Piedmont and Valle d’Aosta Registry (PARALS) established in 1995, and the Emilia-Romagna Registry (ERRALS) established in 2009, and also the directories of death certificates, hospital discharge records, and drug prescriptions, focusing on the administration of riluzole, the only drug approved for ALS treatment. We included as participants ‘definite’ and ‘probable’ ALS cases as for the El Escorial criteria [19]. Identified cases were also tested for repeat expansions in the *C9orf72* gene. We identified a referent population through random selection of four controls from the National Health Service directory of the residents in the study provinces, matched by sex, age (+/− 5 years), and province of residence to each case. We recruited study participants at the Neurology Departments of the study area hospitals (for cases only) or by regular-mail. In the latter case, along with the invitation letter and the information leaflet with a description of the aim of the study, we enclosed the informed consent form, the questionnaire, and a prepaid return envelope. We implemented a self-administered questionnaire that has been tailored to assess the role of several occupational and environmental factors, including residential history, and occupational and environmental exposures to metals and chemicals, particularly pesticides and solvents.

We estimated the risk of ALS by calculating the disease odds ratio (OR) and 95% confidence intervals (CI) in crude and adjusted unconditional logistic regression models. In the final multivariable model, we included sex, age (as continuous variable), and educational attainment (in four categories: primary school or less, middle school, high school, and college or higher) as potential confounding factors. To code occupational history, we used the European and International standard classification of occupation (ISCO), which classifies jobs with respect to the type of work performed (http://www.ilo.org/public/english/bureau/stat/isco/). We also performed stratified analysis by sex and geographic area (Southern versus Northern Italy, i.e., Catania province versus the remaining provinces). We used Stata Software (v16.1, Stata Corp, TX, 2019, USA) for all data analysis.

## 3. Results

Table 1 summarizes the characteristics of the 230 study participants, including 95 (men/women: 51/44) cases and 135 (men/women: 71/64) controls. The positive response rate was 18.9% (230/1218), ranging from 9.7% (40/412) in Sicily to 20.2% (68/337) in Piedmont, and 26.0% (122/469) in Emilia-Romagna. Mean age was 65.8 years (standard deviation: 10.9), with a slightly lower value in controls. Detailed characteristics of non-respondents available for the Emilia-Romagna area are reported in Table S1, showing similar distribution according sex and age of study participants. With regard to educational attainment, a higher proportion of controls graduated at high school or at the university level compared with cases, while the latter show a lower proportion of unmarried and previously married participants. Five and two subjects, respectively, in the cases and controls reported at least one relative within the second grade affected by ALS. In six cases, repeat expansion mutation in the *C9orf72* gene was found, with two of them reporting an ALS diagnosis in the family.

The association of occupational history with disease risk is reported in Table 2. Subjects working in agriculture showed a higher risk (OR = 2.09, 95% CI 0.79–7.59), while for activities in the manufacturing sector, the OR was 1.48 (95% CI 0.80–2.74). Stratified analysis for individual jobs, using technicians and associated work as reference, showed the lowest risk for clerical supporting work, while we found increased risk for those occupied in the armed forces, services and sale work, skilled agricultural work, and housewives.

In particular, history of any occupation related to agriculture showed an increased ALS risk (OR = 2.53, 95% CI 1.15–5.57), with a higher risk for duration of work of 10 years and higher (OR = 2.72, 95% CI 1.02–7.20). As concerns welding activity, we found a slight increased ALS risk, but the estimate was most statistically imprecise due to the very low number of exposed subjects. Concerning the history of any chemical exposure, we found an increased OR of the disease in subjects reporting exposure to metals and metalloids (OR = 4.20, 95% CI 1.88–9.38), particularly mercury (OR = 4.87, 95% CI 0.92–25.80), lead (OR = 3.66, 95% 1.63–9.38), and selenium (OR = 2.54, 95% CI 0.40–16.22). Overall exposure to pesticides showed a somehow increased risk (OR = 1.22, 95% CI 0.63–2.37), with a higher value for fungicides (OR = 1.81, 95% CI 0.70–4.71). Conversely, overall exposure to chemicals and solvents showed an imprecisely increased risk, with higher ORs for exposure to thinners and paint removers (OR = 2.27, 95% CI 1.14–4.54, and 2.01, 95% CI 0.90–4.48, respectively). As regards physical agents, only exposure to electromagnetic fields shows an increased though statistically unstable risk (OR = 1.69, 95% CI 0.70–4.09).

Analysis of residential history (Table 3) indicated that subjects reporting to have lived in the countryside or on a farm had a small increased risk of ALS (OR = 1.36, 95% CI 0.78–2.37), which was slightly higher in those documenting an exposure of 10 years or more (OR = 1.41, 95% CI 0.79–2.51). In regards to other residential factors, those who have ever lived in the proximity of a body water were associated with an increased ALS risk with an OR of 1.83 (95% CI 1.04–3.21). Similarly, we found an increased risk with the proximity of the residence to overhead power lines (OR = 2.41, 95% CI 1.13–5.12). Finally, residential use of pesticides seemed to be possibly associated with decreased risk, either for their use on plants or for animals, with an OR of 0.60 (95% CI 0.33–1.12) and 0.80 (95% CI 0.46–1.37), respectively. Sensitivity analysis after exclusion of subjects reporting a positive family history of ALS yielded substantially comparable results (Tables S2 and S3). Stratified analysis by sex demonstrated comparable results (Tables S4–S7), except for a higher risk in men for working in the agricultural sector, while we found higher risk in women for occupational exposure to electromagnetic fields (Tables S4 and S5). As concerns residential exposure, the increased risk of living in the countryside seemed limited to men only, whereas we found higher risk for living in the proximity of a water body in women than in men, in whom in turn we found a higher risk for residential proximity to overhead power lines (Tables S6 and S7). In stratified analyses according to study area, results were substantially similar (Tables S8–S11), though estimates were less precise for the smaller sample sizes. Finally, we found substantially comparable risk estimates in a stratified analysis, after the exclusion of cases carrying the *C9orf72* gene mutation (Tables S12 and S13).

## 4. Discussion

In this population-based case-control study on the environmental and occupational factors and ALS risk, we did not detect an association between occupational history in the service sector and ALS risk, while both agricultural and manufacturing sectors showed a somewhat increased risk, as reported by others [20,21]. In particular, our findings are consistent with the results of previous studies reporting increased risk for subjects working in the agricultural sector and who are occupationally exposed to agricultural chemicals, especially pesticides [22,23,24,25,26,27,28,29]. Additionally, we found a higher risk for subjects with a duration of work in the agricultural sector equal or higher than 10 years compared to < 10 years, suggestive of some dose-response relation. Similarly, occupational exposure to any type of pesticides showed a positive association with disease risk, with higher association for fungicides, as well as for residential living in a rural area, particularly for >10 years. These results may be explained by the fact that subjects that reported to live in a rural area were also those who more likely work in agricultural activities [30,31,32,33]. As a matter of fact, when we further include in the model both occupational sector and living in the countryside, OR slightly decreased for the latter (OR = 1.22, 95% CI 0.68–2.19), as well as for working in a primary sector (OR = 1.89, 95% CI 0.60–5.93), suggesting confounding effect between the two variables. Conversely, residential use of pesticides showed no such association in our study with ALS risk, with reference either to the use of pesticides for plants or to their use for bugs and other animals. We have previously found similar substantial inconclusive findings when we evaluated residential exposure to pesticides based on crop proximity of the house of the subjects, except for an imprecise increase for citrus orchards in the Sicily region [34].

In our study, some specific occupations, other than agricultural workers, appeared to be at higher risk of ALS. We found that subjects that have worked in the armed forces have increased ALS risk, despite the fact that an increased risk for men who attended military service was not noted. The hypothesis of a positive association between military service and ALS was originally generated by the observation of an unexpectedly high disease risk in Gulf War Veterans [35,36,37,38]. Military service has been associated with increased disease risk in some studies [39,40,41,42,43], though not in all [44,45,46,47]. Despite factors underlying a possible excess of ALS among workers in armed forces remain unidentified, several factors have been suggested including strenuous physical exertion, poor sleep, trauma, psychological stress, and exposure to chemicals including heavy metals or pesticides [39,48,49].

As regards to the manufacturing sector, we found some evidence of a positive association for subjects reporting a professional exposure to welding activities, and for the occupational use of heavy metals, particularly mercury, lead, and selenium. This is consistent with previous studies that suggested an increased ALS risk for working as welder [24,50,51,52,53,54], as well as the use of heavy metals [22,24,27,47,55,56]. Interestingly, when performing analysis of heavy metals in biological samples, higher levels of lead and other heavy metals were found in ALS cases compared to controls [57,58,59]. In addition, selenium exposure has been also associated with increased incidence and mortality of ALS after ingestion of its hexavalent inorganic form through drinking water [16,60]. Similarly, higher concentrations of inorganic selenium (i.e., selenite) in cerebrospinal fluid of newly-diagnosed ALS cases have been associated with increased disease risk [61]. Conversely, as concern overall solvent and chemical exposure during working activity, we found little evidence of a positive association, as reported in previous studies [24,25,27,55,62].

Noteworthy, in our study, occupational exposure to electromagnetic fields (EMF) suggested a positive association with ALS risk, but this was not true for exposure to electric and electronic equipment. Similar positive association for EMF exposure was shown in some studies [23,24,50,63,64,65]. In addition, we found a suggestive positive association between EMF exposure and ALS risk when we assessed the proximity to overhead power lines. Similar positive though limited association for residential exposure to EMF and ALS was found in one previous study carried in Swiss [66], but not in others studies [67,68,69], including a previous study of ours carried out in part of the study area, namely the Emilia-Romagna and Sicily regions [70].

Another environmental risk factor that has been suggested is the environmental exposure to the beta-methylamino-L-alanine (BMAA) neurotoxic, which is produced by cyanobacteria. This hypothesis has been originally proposed after an occurrence of a very high incidence of ALS and other neurodegenerative diseases occurred in the island of Guam [71]. This seemed to be linked to the dietary habits of the native Chamorro population [72]. In particular, it has been proposed that a biomagnification of the BMAA neurotoxin was ingested through the intake of seeds of the cycad plant, possibly contaminated by the BMAA neurotoxin released by the cyanobacteria that lived symbiotically in their roots [73,74]. Another hypothesis includes the aerosolubilization of BMAA neurotoxin from a cyanobacteria algal bloom [75]. Our results are consistent with these findings, which linked ALS with living near lakes with the presence of algal bloom [76,77]. However, we did not collect water samples and assess the presence of contamination, thus hampering a further assessment of the linkage with a specific biological agent. Finally, we did not find any convincing association in our study between holding an animal and ALS risk, which is consistent with some reports [78,79], but not with others [80,81,82].

A few limitations of our study should be pointed out. The small sample size has affected the precision of the risk estimates. In addition, the low response rate may have been a source of selection bias in the referent population. However, when we compared the characteristics of participants to those of the general population, we found a substantially similar distribution. In particular, the distribution of educational level in controls is comparable to the Italian population over 35 years based on 2011 census data (http://dati-censimentopopolazione.istat.it/Index.aspx), thus suggesting little evidence of selection bias. Secondly, age and sex distribution of non-respondents, which was available for the Emilia-Romagna participants, was similar to that of responders, thus reducing the likelihood of selection bias. In addition, we may have missed cases that were already dead or with severe symptoms hampering participation in the survey, which could have been characterized by a different distribution of exposure under the study compared with the cases we could recruit. However, in the study invitation, we already expected that the caregiver or other relative could fill out the questionnaire in case of high severity of the disease, thus limiting the loss of cases for that reason.

We also acknowledge that the questionnaire relied on self-report for the exposure assessment, and that this may have caused some recall bias, especially when seeking historical information about exposure to contaminants and other factors that the cases could have perceived as associated with disease risk, with higher and differential reporting compared to controls. In addition, the reliability and precision in recalling historical exposures may be challenged, especially when it occurred several years before enrolment. However, a validation study evaluating the performance of the geographical information system (GIS) for the assessment of residential pesticide exposure in two regions (Emilia-Romagna and Sicily) showed that comparable exposure classification can be derived by questionnaire-based and GIS-based assessments [83], thus reassuring us about the validity of the exposure assessment used in the present study. Finally, due to the nonexperimental study design, we cannot entirely rule out the presence of residual confounding variable. In particular, though we did not include socioeconomic status as a potential confounder, we considered educational attainment. However, the potential for a confounding variable of socioeconomic status is likely to be limited given the recent results of an Irish study, which considered the social deprivation index and population density of a residential area [84].

Strengths of this study include its population-based design, since we recruited controls from the general population. In addition, the study area encompassed different Italian regions, located in the Northern and the Southern part of the country, and was expected to be characterized by different life-style and environmental exposures and possibly genetic background, factors that might modify disease susceptibility [85] and that we investigated through subgroup analyses. Finally, we were able to perform sensitivity analysis by excluding subjects reporting a history of ALS in their relatives [86] in order to assess the specific role of environmental risk factors for sporadic ALS.

## 5. Conclusions

This case-control study suggests that some environmental risk factors may play a role in the onset of sporadic ALS. In particular, having an occupation in the agricultural sector, especially with a long duration of the working activity as well as occupational exposure to some chemicals, such as heavy metals and selenium, might increase ALS risk. In addition, a slightly positive association emerged for either occupational or environmental exposure to electromagnetic fields, and for occupational pesticide exposure only. Finally, positive association with environmental exposure to either chemical or biological agents as a result of living in proximity of water bodies is also suggested, which is in line with the possibility that cyanobacterial exposure might be associated with disease risk. These positive findings, however, need to be interpreted with caution, given that there are some study limitations, such as the statistical precision of the estimates due to small sample size and few exposed subjects, and that there are still uncertainties that exist about the mechanisms involved in ALS etiology.

## Figures and Tables

**Table 1:** Characteristics of study population.

Characteristics	Cases *n* (%)	Controls *n* (%)	Total *n* (%)
**All Subjects**	95 (100)	135 (100)	230 (100)
**Sex**			
Men	51 (53.7)	71 (52.6)	122 (53.0)
Women	44 (46.3)	64 (47.4)	108 (47.0)
**Age**			
Mean (SD) Years	64.9 (11.7)	66.5 (10.3)	65.8 (10.9)
<65 years	46 (48.4)	56 (41.5)	102 (44.3)
≥65 years	49 (51.6)	79 (58.5)	128 (55.6)
**Educational Attainment**			
Primary School or Less	40 (42.1)	47 (34.8)	87 (37.8)
Middle School	24 (25.3)	28 (20.7)	52 (22.6)
High School	23 (24.2)	42 (31.1)	65 (28.3)
College or Higher	8 (8.4)	18 (13.4)	26 (11.3)
**Marital Status**			
Married	64 (67.4)	101 (74.8)	165 (71.7)
Unmarried	9 (9.5)	8 (5.9)	17 (7.4)
Previously Married	22 (23.2)	26 (19.3)	48 (20.9)
**Occupational Sector**			
Agriculture Sector	9 (9.5)	8 (5.9)	17 (7.4)
Manufacturing Sector	48 (50.5)	55 (40.7)	103 (44.8)
Service Sector	38 (40.0)	72 (53.3)	110 (47.8)
**Occupational Category**			
Armed Forces	2 (2.1)	2 (1.5)	4 (1.7)
Managers	2 (2.1)	3 (2.2)	5 (2.2)
Professionals/Intellectuals	5 (5.3)	10 (7.4)	15 (6.5)
Technicians and Associate Workers	18 (18.9)	22 (16.3)	40 (17.4)
Clerical Support Workers	5 (5.3)	24 (17.8)	29 (12.6)
Services and Sales Workers	13 (13.7)	10 (7.4)	23 (10.0)
Skilled Agricultural Workers	9 (9.5)	8 (5.9)	16 (7.4)
Craft and Related Trades Workers	26 (27.4)	26 (19.3)	52 (22.6)
Plant and Machine Operator Workers	4 (4.2)	7 (5.2)	11 (4.8)
Retired or Other Occupation	5 (5.3)	18 (13.3)	23 (10.0)
Housewives	6 (6.3)	5 (3.7)	11 (4.8)
**Military Service (Men only)**			
Yes	32 (62.7)	46 (64.8)	78 (63.9)
No	19 (37.3)	25 (35.2)	44 (36.1)
**Province of Residence**			
Catania	19 (20.0)	21 (15.6)	40 (17.4)
Modena	29 (30.5)	47 (34.8)	76 (33.0)
Reggio Emilia	13 (13.7)	33 (24.4)	46 (20.0)
Novara	34 (37.8)	34 (25.2)	68 (29.6)
**ALS Cases in the Family**			
Yes	5 (5.3)	2 (1.5)	7 (3.0)
No	90 (94.7)	133 (98.5)	233 (97.0)

**Table 2:** Odds ratio (OR) with 95% confidence interval (CI) of ALS risk according to occupational history and exposure.

Questionnaire Section	Cases (y/n)	Controls (y/n)	OR ^a^	OR ^b^	(95% CI)
**Occupational History**					
**Working Sector**					
Agriculture Working Sector	9	8	2.13	2.09	(0.79–7.54)
Manufacturing Working Sector	48	55	1.68	1.48	(0.80–2.74)
Service Working Sector	38	72	Ref.	Ref.	-
**Occupational Category**					
Armed Forces	2	2	1.22	1.75	(0.20–15.04)
Managers	2	3	0.81	0.94	(0.14–6.48)
Professionals/Intellectuals	5	10	0.61	0.76	(0.18–3.18)
Technicians and Associate Workers	18	22	Ref.	Ref.	-
Clerical Support Workers	5	24	0.25	0.29	(0.09–0.94)
Services and Sales Workers	13	10	1.59	1.67	(0.55–5.07)
Skilled Agricultural Workers	9	8	1.38	1.52	(0.42–5.45)
Craft and related trades workers	26	26	1.22	1.06	(0.43–2.62)
Plant and Machine Operator Workers	4	7	0.70	0.57	(0.14–2.39)
Retired or other occupation	5	18	0.34	0.32	(0.09–1.13)
Housewives	6	5	1.47	1.37	(0.32–5.78)
**Previous Agricultural Work**	21/74	13/122	2.66	2.53	(1.15–5.57)
No agricultural Work	74	122	Ref.	Ref.	
Duration of Work 1–10 years	8	5	2.64	2.26	(0.69–7.41)
Duration of Work ≥10 years	13	8	2.68	2.72	(1.02–7.20)
**Work as Welder**	7/88	5/130	2.07	1.67	(0.49–5.66)
**Presence of the Photocopier at Work**	23/72	44/91	0.66	0.66	(0.33–1.33)
**Military Service** ^c^	32/19	46/25	0.92	0.82	(0.37–1.82)
**Occupational Exposure to Toxic Agents**					
**Metals/Metalloids**	25/70	13/122	3.35	4.20	(1.88–9.38)
Lead	23/72	13/122	3.00	3.66	(1.63–8.20)
Mercury	6/89	2/133	4.48	4.87	(0.92–25.80)
Selenium	3/92	2/133	2.17	2.54	(0.40–16.22)
Cadmium	2/93	2/133	1.43	1.79	(0.23–13.73)
**Overall Pesticides**	21/74	25/110	1.25	1.22	(0.63–2.37)
Insecticides	21/74	23/112	1.36	1.34	(0.69–2.64)
Herbicides	12/83	13/122	1.36	1.37	(0.58–3.21)
Fungicides	11/84	9/126	1.83	1.81	(0.70–4.71)
**Overall Chemicals/Solvents**	44/51	50/85	1.47	1.46	(0.82–2.61)
Oil Paints	9/86	9/126	1.47	1.47	(0.53–4.05)
Thinners	28/67	22/113	2.15	2.27	(1.14–4.54)
Paint Removers	18/77	15/120	1.87	2.01	(0.90–4.48)
Paints	20/75	19/116	1.63	1.73	(0.80–3.75)
Adhesives	13/82	18/117	1.03	1.07	(0.48–2.38)
Print inks and Dyes	11/84	15/120	1.05	1.05	(0.45–2.47)
Lubricating oils	18/77	19/116	1.43	1.50	(0.69–3.27)
Refrigerants, Antifreezes, and Cooling Liquids	7/88	13/122	0.75	0.76	(0.27–2.11)
Degreasing Agents	11/84	16/119	0.97	0.97	(0.42–2.26)
Solvents (e.g., toluene, xylene)	9/86	8/127	1.66	1.68	(0.59–4.78)
Dry Clean Products	3/92	9/126	0.46	0.42	(0.11–1.62)
**Anesthetic Gas**	1/94	6/129	0.23	0.24	(0.03–2.01)
**Overall Electro-magnetic Factors**	29/66	37/98	1.16	1.11	(0.59–2.08)
Electric and Electronic Equipment	23/72	35/100	0.91	0.85	(0.44–1.62)
Electromagnetic Fields	13/82	12/123	1.62	1.69	(0.70–4.09)

^a^ Crude model; ^b^ Model adjusted by sex, age, and educational attainment; ^c^ In men only.

**Table 3:** Odds ratio (OR) with 95% confidence interval (CI) of ALS risk according residential information and non-occupational use of pesticides.

Questionnaire Section	Cases (y/n)	Controls (y/n)	OR ^a^	OR ^b^	(95% CI)
**Residential Information**					
Ever Lived in the Countryside or Had a Farm	42/53	47/88	1.48	1.36	(0.78–2.37)
Duration ≥10 years	37/58	40/95	1.52	1.41	(0.79–2.51)
Ever Lived Less than 3 km from Water Bodies	41/54	40/95	1.80	1.83	(1.04–3.21)
Having Lived near Waste Incinerator	6/89	5/130	1.75	1.72	(0.50–5.87)
Having Lived near Waste Disposal Site	7/88	15/120	0.64	0.57	(0.22–1.51)
Having Lived near Overhead Power Lines	21/74	14/121	2.45	2.41	(1.13–5.12)
**Use of Pesticides** (**not Occupational**)					
Have Pets Living Indoor	28/67	42/93	0.93	0.84	(0.46–1.53)
Ever Had Pets Living Indoor	72/23	103/32	0.97	1.07	(0.57–2.00)
Use of Flea and Tick Products on Pets	40/55	53/82	1.13	1.10	(0.64–1.92)
Overall Pesticides Use on Plants	21/74	43/92	0.61	0.60	(0.33–1.12)
Pesticides Use on Meadow	11/84	24/111	0.61	0.60	(0.27–1.31)
Pesticides Use on Outdoor Plants	16/79	31/104	0.68	0.67	(0.34–1.33)
Pesticides Use on Indoor Plants	3/92	6/129	0.70	0.77	(0.18–3.25)
Overall Pesticides Use on Animals	40/55	68/67	0.72	0.80	(0.46–1.37)
Pesticides Use on Flying Bugs	34/61	62/73	0.66	0.71	(0.41–1.24)
Pesticides Use on Ground Bugs	21/74	38/97	0.72	0.81	(0.43–1.53)
Pesticides Use on Rodents and Rats	6/89	19/111	0.41	0.45	(0.17–1.18)
Pesticides Use on Other Animals	17/78	25/110	0.96	0.94	(0.47–1.88)
Bug Disinfection in the Residence	6/89	8/127	1.07	1.17	(0.38–3.59)
History of Pesticides Use by the Municipality	40/55	49/86	1.28	1.15	(0.66–2.01)

^a^ Crude model; ^b^ Model adjusted by sex, age, and educational attainment.

## Supplementary Materials

The following are available online at https://www.mdpi.com/1660-4601/17/8/2882/s1, Table S1. Characteristics of non-responders for Emilia-Romagna region. Table S2. Odds ratio (OR) with 95% confidence interval (CI) of ALS risk according to occupational information without subjects with family history of ALS. Table S3. Odds ratio (OR) with 95% confidence interval (CI) of ALS risk according residential information and non-occupational use of pesticides without subjects with family history of ALS. Table S4. Odds ratio (OR) with 95% confidence interval (CI) of ALS risk according to occupational history and exposure in men. Table S5. Odds ratio (OR) with 95% confidence interval (CI) of ALS risk according to occupational history and exposure in women. Table S6. Odds ratio (OR) with 95% confidence interval (CI) of ALS risk according residential information and non-occupational use of pesticides in men. Table S7. Odds ratio (OR) with 95% confidence interval (CI) of ALS risk according residential information and non-occupational use of pesticides in women. Table S8. Odds ratio (OR) with 95% confidence interval (CI) of ALS risk according to occupational history and exposure in Northern Italy provinces (Modena, Novara and Reggio Emilia). Table S9. Odds ratio (OR) with 95% confidence interval (CI) of ALS risk according to occupational history and exposure in Southern Italy province (Catania). Table S10. Odds ratio (OR) with 95% confidence interval (CI) of ALS risk according residential information and non-occupational use of pesticides in Northern Italy provinces (Modena, Novara and Reggio Emilia). Table S11. Odds ratio (OR) with 95% confidence interval (CI) of ALS risk according residential information and non-occupational use of pesticides in Southern Italy province (Catania). Table S12. Odds ratio (OR) with 95% confidence interval (CI) of ALS risk according to occupational information without carriers of *C9orf72* mutation. Table S13. Odds ratio (OR) with 95% confidence interval (CI) of ALS risk according to leisure activities and other lifestyle factors without carriers of *C9orf72* mutation.
