# Supplementary material for: Environmental and Occupational Risk Factors of Amyotrophic Lateral Sclerosis: A Population-Based Case-Control Study
Source: Int J Environ Res Public Health. 2020 Apr 22;17(8):2882. doi: 10.3390/ijerph17082882 (PMC7216189; doi:10.3390/ijerph17082882)
Supplement: Supplementary file 1 [file ijerph-17-02882-s001.pdf]

International Journal of
*Environmental Research and Public Health*

MDPI

**Table S1.** Characteristics of non-responders for Emilia-Romagna region.

| | Cases n (%) | Controls n (%) | Total n (%) |
|---|---|---|---|
| **Modena** | | | |
| Total Questionnaires | 82 (100) | 197 (100) | 279 |
| Non-responders | 53 (65) | 150 (76) | 203 (73) |
| Age - Mean (SD) | 71.9 (13.2) | 71.5 (13.6) | 71.6 (13.5) |
| **Sex** | | | |
| Men | 29 (55) | 84 (56) | 113 (56) |
| Women | 24 (45) | 66 (44) | 90 (44) |
| **Reggio Emilia** | | | |
| Total Questionnaires | 57 (100) | 133 (100) | 190 |
| Non-responders | 44 (77) | 100 (75) | 144 (76) |
| Age - Mean (SD) | 70.6 (10.9) | 69.5 (10.9) | 69.8 (10.9) |
| **Sex** | | | |
| Men | 22 (50) | 56 (56) | 78 (54) |
| Women | 22 (50) | 44 (44) | 66 (46) |

n: number of subjects; SD: standard deviation.

**Table S2.** Odds ratio (OR) with 95% confidence interval (CI) of ALS risk according to occupational information without subjects with family history of ALS.

| Questionnaire Section | Cases (y/n) | Controls (y/n) | OR[a] | OR[b] | (95% CI) |
|---|---|---|---|---|---|
| **Occupational History** | | | | | |
| **Working Sector** | | | | | |
| Agriculture Working Sector | 9 | 8 | 2.19 | 2.04 | (0.67-6.18) |
| Manufacturing Working Sector | 45 | 55 | 1.59 | 1.40 | (0.75-2.61) |
| Service Working Sector | 36 | 70 | Ref. | Ref. | - |
| **Occupational Category** | | | | | |
| Armed Forces | 2 | 2 | 1.29 | 1.98 | (0.23-17.15) |
| Managers | 2 | 3 | 0.86 | 0.99 | (0.14-6.78) |
| Professionals/Intellectuals | 5 | 9 | 0.72 | 0.99 | (0.23-4.36) |
| Technicians and Associate Workers | 17 | 22 | Ref. | Ref. | - |
| Clerical Support Workers | 4 | 23 | 0.23 | 0.27 | (0.07-0.94) |
| Services and Sales Workers | 13 | 10 | 1.68 | 1.65 | (0.54-5.06) |
| Skilled agricultural workers | 9 | 8 | 1.46 | 1.47 | (0.40-5.33) |
| Craft and Related Trades Workers | 24 | 26 | 1.19 | 1.00 | (0.40-2.50) |
| Plant and Machine Operator Workers | 4 | 7 | 0.74 | 0.59 | (0.14-2.46) |
| Retired or Other Occupation | 5 | 18 | 0.36 | 0.32 | (0.09-1.15) |
| Housewives | 5 | 5 | 1.29 | 1.19 | (0.27-5.26) |
| **Previous Agricultural Work** | 20/70 | 13/120 | 2.64 | 2.50 | (1.12-5.57) |
| No Agricultural Work | 70 | 120 | Ref. | Ref. | |
| Duration of Work 1-10 years | 8 | 5 | 2.74 | 2.38 | (0.72-7.81) |
| Duration of Work ≥10 years | 12 | 8 | 2.57 | 2.58 | (0.95-7.00) |
| **Work as Welder** | 7/83 | 5/128 | 2.16 | 1.83 | (0.54-6.24) |
| **Presence of the Photocopier at Work** | 21/69 | 43/90 | 0.64 | 0.68 | (0.33-1.38) |
| **Military service**[c] | 31/16 | 46/25 | 1.05 | 0.97 | (0.43-2.19) |
| **Occupational Exposure to Toxic Agents** | | | | | |
| **Metals/Metalloids** | 24/66 | 13/120 | 3.36 | 4.43 | (1.95-10.04) |
| Lead | 22/68 | 13/120 | 2.99 | 3.82 | (1.68-8.69) |
| Mercury | 5/85 | 2/131 | 3.85 | 4.36 | (0.92-25.05) |

| | | | | | |
|---|---|---|---|---|---|
| Selenium | 3/87 | 2/131 | 2.26 | 2.62 | (0.41-16.70) |
| Cadmium | 2/88 | 2/131 | 1.49 | 1.87 | (0.24-14.45) |
| **Overall Pesticides** | 20/70 | 24/109 | 1.30 | 1.29 | (0.65-2.55) |
| Insecticides | 20/70 | 22/111 | 1.44 | 1.43 | (0.72-2.85) |
| Herbicides | 11/79 | 13/120 | 1.29 | 1.33 | (0.56-3.18) |
| Fungicides | 11/79 | 8/125 | 2.18 | 2.22 | (0.83-5.97) |
| **Overall Chemicals/Solvents** | 42/48 | 50/83 | 1.45 | 1.48 | (0.82-2.67) |
| Oil paints | 9/81 | 9/124 | 1.53 | 1.61 | (0.58-4.47) |
| Thinners | 27/63 | 22/111 | 2.16 | 2.38 | (1.18-4.81) |
| Paint Removers | 17/73 | 15/118 | 1.83 | 2.04 | (0.90-4.63) |
| Paints | 19/71 | 19/114 | 1.61 | 1.84 | (0.84-4.03) |
| Adhesives | 13/77 | 18/115 | 1.08 | 1.12 | (0.50-2.50) |
| Print Inks and Dyes | 10/80 | 15/118 | 0.98 | 0.99 | (0.41-2.38) |
| Lubricating Oils | 18/72 | 19/114 | 1.50 | 1.68 | (0.76-3.70) |
| Refrigerants, Antifreezes, and Cooling Liquids | 7/83 | 13/120 | 0.78 | 0.84 | (0.30-2.34) |
| Degreasing Agents | 11/79 | 16/117 | 1.02 | 1.03 | (0.44-2.40) |
| Solvents (e.g., toluene, xylene) | 8/82 | 8/125 | 1.52 | 1.60 | (0.55-4.64) |
| Dry Clean Products | 3/87 | 9/124 | 0.48 | 0.43 | (0.11-1.69) |
| **Anesthetic Gas** | 1/89 | 6/127 | 0.24 | 0.31 | (0.03-2.78) |
| **Overall Electro-magnetic Factors** | 29/64 | 37/96 | 1.05 | 1.01 | (0.53-1.91) |
| Electric and Electronic Equipment | 21/69 | 35/98 | 0.85 | 0.81 | (0.42-1.57) |
| Electromagnetic Fields | 11/79 | 12/121 | 1.40 | 1.50 | (0.60-3.73) |

[a]Crude model; [b]Model adjusted by sex, age, and educational attainment; [c]In men only.

**Table S3.** Odds ratio (OR) with 95% confidence interval (CI) of ALS risk according residential information and non-occupational use of pesticides without subjects with family history of ALS.

| Questionnaire Section | Cases (y/n) | Controls (y/n) | OR[a] | OR[b] | (95% CI) |
|---|---|---|---|---|---|
| **Residential Information** | | | | | |
| Ever Lived in the Countryside or Had a Farm | 42/49 | 46/87 | 1.58 | 1.42 | (0.81-2.51) |
| Duration ≥10 years | 36/54 | 39/94 | 1.61 | 1.47 | (0.81-2.64) |
| Ever Lived less than 3 km from Water Bodies | 39/51 | 38/95 | 1.91 | 1.97 | (1.11-3.50) |
| Having Lived near Waste Incinerator | 6/84 | 5/128 | 1.83 | 1.81 | (0.53-6.20) |
| Having Lived near Waste Disposal Site | 6/84 | 15/118 | 0.56 | 0.50 | (0.18-1.38) |
| Having Lived near Overhead Power Lines | 19/71 | 14/119 | 2.27 | 2.29 | (1.06-4.93) |
| **Use of Pesticides (not Occupational)** | | | | | |
| Have Pets Living Indoor | 27/63 | 41/92 | 0.96 | 0.89 | (0.49-1.64) |
| Ever had Pets Living Indoor | 68/22 | 101/32 | 0.98 | 1.09 | (0.58-2.07) |
| Use of Flea and Tick Products on Pets | 39/51 | 52/81 | 1.19 | 1.20 | (0.68-2.11) |
| Overall pesticides use on plants | 21/69 | 41/92 | 0.68 | 0.68 | (0.37-1.27) |
| Pesticides Use on Meadow | 11/79 | 23/110 | 0.67 | 0.67 | (0.30-1.47) |
| Pesticides Use on Outdoor Plants | 16/74 | 29/104 | 0.78 | 0.78 | (0.39-1.56) |
| Pesticides Use on Indoor Plants | 3/87 | 6/127 | 0.73 | 0.74 | (0.17-3.17) |
| Overall Pesticides Use on Animals | 39/51 | 66/67 | 0.78 | 0.86 | (0.50-1.50) |
| Pesticides Use on Flying Bugs | 33/57 | 60/73 | 0.70 | 0.77 | (0.44-1.36) |
| Pesticides Use on Ground Bugs | 20/70 | 37/96 | 0.74 | 0.82 | (0.43-1.56) |
| Pesticides Use on Rodents and Rats | 6/84 | 18/115 | 0.46 | 0.50 | (0.19-1.34) |
| Pesticides Use on Other Animals | 16/74 | 25/108 | 0.93 | 0.94 | (0.46-1.90) |
| Bug Disinfection in the Residence | 6/84 | 8/125 | 1.12 | 1.19 | (0.38-3.66) |
| History of Pesticides Use by the Municipality | 37/53 | 48/85 | 1.24 | 1.13 | (0.64-1.98) |

[a]Crude model; [b]Model adjusted by sex, age, and educational attainment.

**Table S4.** Odds ratio (OR) with 95% confidence interval (CI) of ALS risk according to occupational history and exposure in men.

| Questionnaire Section | Cases (y/n) | Controls (y/n) | OR[a] | OR[b] | (95% CI) |
|---|---|---|---|---|---|
| **Occupational History** | | | | | |
| **Working Sector** | | | | | |
| Agriculture Working Sector | 4 | 1 | 9.54 | 10.87 | (1.04-113.42) |
| Manufacturing Working Sector | 34 | 39 | 2.08 | 1.74 | (0.72-4.21) |
| Service Working Sector | 13 | 31 | Ref. | Ref. | - |
| **Occupational Category** | | | | | |
| Armed Forces | 2 | 2 | 1.23 | 2.47 | (0.24-25.27) |
| Managers | 1 | 1 | 1.23 | 0.71 | (0.04-14.39) |
| Professionals/Intellectuals | 1 | 2 | 0.62 | 1.04 | (0.07-16.35) |
| Technicians and Associate Workers | 13 | 16 | Ref. | Ref. | - |
| Clerical Support Workers | 4 | 14 | 0.35 | 0.41 | (0.10-1.70) |
| Services and sales workers | 3 | 5 | 0.74 | 0.62 | (0.12-3.30) |
| Skilled Agricultural Workers | 4 | 1 | 4.92 | 5.66 | (0.53-60.03) |
| Craft and Related Trades Workers | 19 | 16 | 1.46 | 1.06 | (0.36-3.11) |
| Plant and Machine Operator Workers | 2 | 7 | 0.35 | 0.21 | (0.03-1.35) |
| Retired or Other Occupation | 2 | 7 | 0.33 | 0.25 | (0.04-1.73) |
| Housewives | - | - | - | - | |
| **Previous Agricultural Work** | 14/37 | 5/66 | 4.99 | 4.46 | (1.44-13.85) |
| No Agricultural Work | 37 | 166 | Ref. | Ref. | |
| Duration of Work 1-10 years | 6 | 4 | 2.68 | 2.33 | (0.59-9.18) |
| Duration of Work ≥10 years | 8 | 1 | 14.27 | 13.08 | (1.53-11.69) |
| **Work as Welder** | 6/45 | 5/66 | 1.76 | 1.32 | (0.32-4.73) |
| **Presence of the Photocopier at Work** | 17/34 | 27/44 | 0.81 | 0.85 | (0.34-2.11) |
| **Military Service**[c] | 32/19 | 46/25 | 0.92 | 0.82 | (0.37-1.82) |
| **Occupational Exposure to Toxic Agents** | | | | | |
| **Metals/Metalloids** | 22/29 | 11/60 | 4.14 | 5.72 | (2.23-14.70) |
| Lead | 20/31 | 11/60 | 3.52 | 4.73 | (1.85-12.12) |
| Mercury | 6/45 | 1/70 | 9.33 | 9.47 | (1.05-85.45) |
| Selenium | 3/48 | 1/70 | 4.37 | 5.39 | (0.50-57.57) |
| Cadmium | 2/49 | 2/69 | 1.41 | 2.03 | (0.25-16.42) |
| **Overall Pesticides** | 14/37 | 12/59 | 1.86 | 1.83 | (0.74-4.54) |
| Insecticides | 14/37 | 11/60 | 2.06 | 2.04 | (0.81-5.15) |
| Herbicides | 8/43 | 8/63 | 1.47 | 1.52 | (0.51-4.62) |
| Fungicides | 8/43 | 5/66 | 2.46 | 2.69 | (0.79-9.21) |
| **Overall Chemicals/Solvents** | 32/19 | 35/36 | 1.73 | 1.75 | (0.81-3.76) |
| Oil Paints | 9/42 | 8/53 | 1.69 | 1.73 | (0.59-5.06) |
| Thinners | 25/26 | 16/55 | 3.31 | 4.14 | (1.73-9.89) |
| Paint Removers | 18/33 | 13/58 | 2.43 | 2.79 | (1.16-6.71) |
| Paints | 20/31 | 18/53 | 1.90 | 2.00 | (0.89-4.49) |
| Adhesives | 8/43 | 14/57 | 0.76 | 0.77 | (0.28-2.10) |
| Print Inks and Dyes | 7/44 | 9/62 | 1.10 | 1.10 | (0.37-3.27) |
| Lubricating Oils | 17/34 | 18/53 | 1.47 | 1.53 | (0.67-3.50) |
| Refrigerants, Antifreezes, and Cooling liquids | 7/44 | 12/59 | 0.78 | 0.81 | (0.28-2.35) |
| Degreasing Agents | 8/43 | 10/61 | 1.13 | 1.23 | (0.42-3.56) |
| Solvents (e.g., toluene, xylene) | 7/44 | 7/64 | 1.45 | 1.31 | (0.40-4.29) |
| Dry Clean Products | 1/50 | 6/65 | 0.22 | 0.20 | (0.02-1.79) |
| **Anesthetic Gas** | 1/50 | 5/66 | 0.26 | 0.34 | (0.03-3.30) |
| **Overall Electro-magnetic Factors** | 20/31 | 26/45 | 1.12 | 0.99 | (0.43-2.27) |
| Electric and Electronic equipment | 16/35 | 24/47 | 0.90 | 0.76 | (0.32-1.80) |
| Electromagnetic Fields | 10/41 | 10/61 | 1.49 | 1.70 | (0.61-4.75) |

[a]Crude model; [b]Model adjusted by age, and educational attainment.

**Table S5.** Odds ratio (OR) with 95% confidence interval (CI) of ALS risk according to occupational history and exposure in women.

| Questionnaire Section | Cases (y/n) | Controls (y/n) | OR[a] | OR[b] | (95% CI) |
|---|---|---|---|---|---|
| **Occupational History** | | | | | |
| **Working Sector** | | | | | |
| Agriculture Working Sector | 5 | 7 | 1.17 | 1.01 | (0.26-3.97) |
| Manufacturing Working Sector | 14 | 14 | 1.44 | 1.32 | (0.54-3.24) |
| Service Working Sector | 25 | 41 | Ref. | Ref. | - |
| **Occupational Category** | | | | | |
| Armed Forces | 0 | 0 | - | - | |
| Managers | 1 | 2 | 0.60 | 0.57 | (0.04-8.96) |
| Professionals/Intellectuals | 4 | 8 | 0.60 | 0.61 | (0.09-3.93) |
| Technicians and Associate Workers | 5 | 6 | Ref. | Ref. | - |
| Clerical Support Workers | 1 | 10 | 0.12 | 0.12 | (0.01-1.29) |
| Services and Sales Workers | 10 | 5 | 2.40 | 2.70 | (0.44-16.74) |
| Skilled Agricultural Workers | 5 | 7 | 0.86 | 0.79 | (0.09-6.75) |
| Craft and Related Trades Workers | 7 | 10 | 0.84 | 0.83 | (0.12-5.67) |
| Plant and Machine Operator Workers | 2 | 0 | - | - | |
| Retired or Other Occupation | 53 | 11 | 0.33 | 0.30 | (0.04-2.38) |
| Housewives | 6 | 5 | 1.44 | 1.42 | (0.24-8.46) |
| **Previous Agricultural Work** | 7/37 | 8/56 | 1.32 | 1.21 | (0.37-4.01) |
| No Agricultural Work | 37 | 56 | Ref. | Ref. | |
| Duration of Work 1-10 years | 2 | 1 | 3.03 | 2.61 | (0.22-30.73) |
| Duration of Work ≥10 years | 5 | 7 | 1.08 | 0.97 | (0.25-3.73) |
| **Work as Welder** | 1/43 | 0/64 | - | - | |
| **Presence of the Photocopier at Work** | 6/38 | 17/47 | 0.44 | 0.40 | (0.12-1.31) |
| **Military Service**[c] | 0/44 | 0/64 | - | - | |
| **Occupational Exposure to Toxic Agents** | | | | | |
| **Metals/Metalloids** | 3/41 | 2/62 | 2.27 | 2.30 | (0.36-14.73) |
| Lead | 3/41 | 2/62 | 2.27 | 2.30 | (0.36-14.73) |
| Mercury | 0/44 | 1/63 | - | - | |
| Selenium | 0/44 | 1/63 | - | - | |
| Cadmium | 0/44 | 0/64 | - | - | |
| **Overall Pesticides** | 7/37 | 13/51 | 0.74 | 0.72 | (0.26-2.01) |
| Insecticides | 7/37 | 12/52 | 0.82 | 0.79 | (0.28-2.24) |
| Herbicides | 4/44 | 5/59 | 1.18 | 1.25 | (0.31-5.03) |
| Fungicides | 3/41 | 4/60 | 1.10 | 0.97 | (0.19-4.91) |
| **Overall Chemicals/Solvents** | 12/32 | 15/49 | 1.23 | 1.25 | (0.47-3.30) |
| Oil Paints | 0/44 | 1/63 | - | - | |
| Thinners | 3/41 | 6/58 | 0.71 | 0.67 | (0.15-2.91) |
| Paint Removers | 0/44 | 2/62 | - | - | |
| Paints | 0/44 | 1/63 | - | - | |
| Adhesives | 5/39 | 4/60 | 1.92 | 2.08 | (0.50-8.73) |
| Print Inks and Dyes | 4/40 | 6/58 | 0.97 | 1.01 | (0.23-4.38) |
| Lubricating Oils | 1/43 | 1/63 | 1.47 | 1.51 | (0.09-25.60) |
| Refrigerants, Antifreezes, and Cooling Liquids | 9/44 | 1/63 | - | - | |
| Degreasing Agents | 3/41 | 6/58 | 0.71 | 0.68 | (0.16-2.95) |
| Solvents (e.g., toluene, xylene) | 2/42 | 1/63 | 3.00 | 3.53 | (0.29-43.08) |
| Dry Clean Products | 2/42 | 3/61 | 0.97 | 0.93 | (0.14-6.04) |
| **Anesthetic Gas** | 0/44 | 1/63 | - | - | |
| **Overall Electro-magnetic Factors** | 9/35 | 11/53 | 1.24 | 1.24 | (0.45-3.40) |
| Electric and Electronic Equipment | 7/37 | 11/53 | 0.91 | 0.91 | (0.32-2.64) |
| Electromagnetic Fields | 3/41 | 2/62 | 2.27 | 2.32 | (0.33-16.25) |

[a]Crude model; [b]Model adjusted by age, and educational attainment.

**Table S6.** Odds ratio (OR) with 95% confidence interval (CI) of ALS risk according residential information and non-occupational use of pesticides in men.

| Questionnaire Section | Cases (y/n) | Controls (y/n) | OR[a] | OR[b] | (95% CI) |
|---|---|---|---|---|---|
| **Residential Information** | | | | | |
| Ever Lived in the Countryside or Had a Farm | 22/29 | 19/52 | 2.08 | 1.87 | (0.85-4.14) |
| Duration ≥ 10 years | 20/31 | 15/56 | 2.41 | 2.24 | (0.98-5.12) |
| Ever Lived less than 3 km from Water Bodies | 22/29 | 25/46 | 1.40 | 1.48 | (0.68-3.19) |
| Having Lived near Waste Incinerator | 4/47 | 3/68 | 1.93 | 2.05 | (0.42-10.04) |
| Having Lived near Waste Disposal Site | 2/49 | 5/66 | 0.54 | 0.38 | (0.06-2.21) |
| Having Lived near Overhead Power Lines | 14/37 | 8/63 | 2.98 | 3.04 | (1.13-8.18) |
| **Use of Pesticides (not Occupational)** | | | | | |
| Have Pets Living Indoor | 13/38 | 18/53 | 1.01 | 0.81 | (0.33-1.99) |
| Ever had Pets Living Indoor | 41/10 | 53/18 | 1.39 | 1.83 | (0.72-4.65) |
| Use of Flea and Tick Products on Pets | 21/30 | 29/42 | 1.01 | 0.95 | (0.44-2.04) |
| Overall Pesticides Use on Plants | 13/38 | 20/51 | 0.87 | 0.86 | (0.37-1.99) |
| Pesticides Use on Meadow | 7/44 | 9/62 | 1.10 | 1.10 | (0.37-3.27) |
| Pesticides Use on Outdoor Pants | 12/39 | 16/55 | 1.06 | 1.09 | (0.45-2.64) |
| Pesticides Use on Indoor Plants | 0/51 | 2/69 | - | - | |
| Overall Pesticides Use on Animals | 20/31 | 35/36 | 0.66 | 0.77 | (0.36-1.65) |
| Pesticides Use on Flying Bugs | 17/34 | 34/37 | 0.54 | 0.60 | (0.28-1.30) |
| Pesticides Use on Ground Bugs | 8/43 | 19/52 | 0.51 | 0.57 | (0.22-1.49) |
| Pesticides Use on Rodents and Rats | 5/46 | 8/63 | 0.86 | 1.09 | (0.32-3.68) |
| Pesticides Use on Other Animals | 7/42 | 16/55 | 0.74 | 0.72 | (0.28-1.85) |
| Bug Disinfection in the Residence | 0/51 | 4/67 | - | - | |
| History of Pesticides Use by the Municipality | 21/30 | 24/47 | 1.37 | 1.10 | (0.50-2.43) |

[a]Crude model; [b]Model adjusted by age, and educational attainment.

**Table S7.** Odds ratio (OR) with 95% confidence interval (CI) of ALS risk according residential information and non-occupational use of pesticides in women.

| Questionnaire Section | Cases (y/n) | Controls (y/n) | OR[a] | OR[b] | (95% CI) |
|---|---|---|---|---|---|
| **Residential Information** | | | | | |
| Ever Lived in the Countryside or Had a Farm | 20/24 | 28/24 | 1.07 | 0.95 | (0.42-2.15) |
| Duration ≥ 10 years | 17/27 | 25/39 | 0.98 | 0.84 | (0.36-1.99) |
| Ever Lived less than 3 km from Water Bodies | 19/25 | 15/49 | 2.48 | 2.41 | (1.03-5.63) |
| Having Lived near Waste Incinerator | 2/42 | 2/62 | 1.48 | 1.44 | (0.19-11.22) |
| Having Lived near Waste Disposal Site | 5/39 | 10/54 | 0.69 | 0.67 | (0.21-2.14) |
| Having Lived near Overhead Power Lines | 7/37 | 6/58 | 1.83 | 1.80 | (0.54-6.07) |
| **Use of Pesticides (not Occupational)** | | | | | |
| Have Pets Living Indoor | 15/29 | 24/40 | 0.86 | 0.82 | (0.36-1.87) |
| Ever had Pets Living Indoor | 3/13 | 50/14 | 0.67 | 0.66 | (0.27-1.61) |
| Use of Flea and Tick Products on Pets | 19/25 | 24/40 | 1.27 | 1.33 | (0.59-2.99) |
| Overall Pesticides Use on Plants | 8/36 | 23/41 | 0.40 | 0.39 | (0.15-1.00) |
| Pesticides Use on Meadow | 4/40 | 15/49 | 0.33 | 0.31 | (0.09-1.03) |
| Pesticides Use on Outdoor Plants | 4/40 | 15/49 | 0.33 | 0.32 | (0.10-1.06) |
| Pesticides Use on Indoor Plants | 3/41 | 4/60 | 1.10 | 1.16 | (0.23-5.79) |
| Overall Pesticides Use on Animals | 20/24 | 33/31 | 0.78 | 0.83 | (0.38-1.82) |
| Pesticides Use on Flying Bugs | 17/27 | 28/36 | 0.81 | 0.86 | (0.38-1.92) |
| Pesticides Use on Ground Bugs | 13/31 | 19/45 | 0.99 | 1.05 | (0.45-2.49) |

| | | | | | |
|---|---|---|---|---|---|
| Pesticides Use on Rodents and Rats | 1/43 | 11/53 | 0.11 | 0.11 | (0.01-0.88) |
| Pesticides Use on Other Animals | 8/36 | 9/55 | 1.36 | 1.43 | (0.49-4.17) |
| Bug Disinfection in the Residence | 6/38 | 4/60 | 2.37 | 2.42 | (0.63-9.31) |
| History of Pesticides Use by the Municipality | 19/25 | 25/39 | 1.19 | 1.14 | (0.52-2.51) |

[a]Crude model; [b]Model adjusted by age, and educational attainment.

**Table S8.** Odds ratio (OR) with 95% confidence interval (CI) of ALS risk according to occupational history and exposure in Northern Italy provinces (Modena, Novara and Reggio Emilia).

| Questionnaire Section | Cases (y/n) | Controls (y/n) | OR[a] | OR[b] | (95% CI) |
|---|---|---|---|---|---|
| **Occupational History** | | | | | |
| **Working Sector** | | | | | |
| Agriculture Working Sector | 8 | 8 | 2.00 | 1.65 | (0.52-5.29) |
| Manufacturing Working Sector | 41 | 52 | 1.58 | 1.31 | (0.66-2.57) |
| Service Working Sector | 27 | 54 | Ref. | Ref. | - |
| **Occupational Category** | | | | | |
| Armed Forces | 1 | 1 | 1.33 | 2.34 | (0.12-45.84) |
| Managers | 1 | 2 | 0.67 | 0.87 | (0.07-10.96) |
| Professionals/Intellectuals | 4 | 6 | 0.89 | 1.45 | (0.26-8.06) |
| Technicians and Associate Workers | 15 | 20 | Ref. | Ref. | - |
| Clerical Support Workers | 4 | 21 | 0.25 | 0.31 | (0.08-1.12) |
| Services and Sales Workers | 13 | 8 | 2.17 | 2.41 | (0.71-8.19) |
| Skilled Agricultural Workers | 8 | 8 | 1.33 | 1.30 | (0.34-5.03) |
| Craft and Related Trades Workers | 22 | 25 | 1.17 | 1.01 | (0.38-2.67) |
| Plant and Machine Operator Workers | 4 | 7 | 0.76 | 0.65 | (0.15-2.81) |
| Retired or Other Occupation | 4 | 15 | 0.36 | 0.33 | (0.08-1.35) |
| Housewives | 0 | 1 | - | - | |
| **Previous Agricultural Work** | 19/57 | 13/101 | 2.59 | 2.30 | (1.02-5.19) |
| No Agricultural Work | 57 | 101 | Ref. | Ref. | |
| Duration of Work 1-10 years | 7 | 5 | 2.48 | 2.07 | (0.60-7.14) |
| Duration of Work ≥ 10 years | 12 | 8 | 2.66 | 2.45 | (0.90-6.66) |
| **Work as Welder** | 7/69 | 4/110 | 2.79 | 2.42 | (0.65-8.98) |
| **Presence of the Photocopier at Work** | 21/55 | 38/76 | 0.76 | 0.80 | (0.37-1.75) |
| **Military Service**[c] | 26/16 | 44/22 | 0.81 | 0.66 | (0.27-1.59) |
| **Occupational Exposure to Toxic Agents** | | | | | |
| **Metals/Metalloids** | 19/57 | 10/104 | 3.47 | 4.26 | (1.71-10.57) |
| Lead | 18/58 | 10/104 | 3.23 | 3.94 | (1.58-9.82) |
| Mercury | 4/72 | 1/113 | 6.28 | 7.03 | (0.74-66.88) |
| Selenium | 3/73 | 1/113 | 4.64 | 5.62 | (0.54-58.10) |
| Cadmium | 2/74 | 2/112 | 1.51 | 2.07 | (0.27-15.96) |
| **Overall Pesticides** | 19/57 | 21/93 | 1.48 | 1.38 | (0.67-2.85) |
| Insecticides | 19/57 | 20/94 | 1.57 | 1.4 | (0.71-3.05) |
| Herbicides | 11/65 | 11/103 | 1.58 | 1.54 | (0.62-3.86) |
| Fungicides | 10/66 | 9/105 | 1.77 | 1.65 | (0.62-4.42) |
| **Overall Chemicals/Solvents** | 36/40 | 43/71 | 1.49 | 1.48 | (0.78-2.82) |
| Oil Paints | 6/70 | 6/108 | 1.54 | 1.74 | (0.51-5.92) |
| Thinners | 24/52 | 18/96 | 2.46 | 2.75 | (1.28-5.92) |
| Paint Removers | 15/61 | 13/101 | 1.91 | 2.29 | (0.95-5.49) |
| Paints | 18/58 | 16/98 | 1.90 | 2.51 | (1.08-5.86) |
| Adhesives | 11/65 | 15/99 | 1.12 | 1.20 | (0.50-2.90) |
| Print Inks and Dyes | 8/68 | 11/103 | 1.10 | 1.15 | (0.42-3.13) |
| Lubricating Oils | 17/59 | 16/98 | 1.76 | 1.97 | (0.84-4.59) |
| Refrigerants, Antifreezes, and Cooling Liquids | 6/70 | 11/103 | 0.80 | 0.80 | (0.26-2.44) |
| Degreasing Agents | 10/66 | 13/101 | 1.18 | 1.15 | (0.46-2.87) |
| Solvents (e.g., toluene, xylene) | 6/70 | 7/107 | 1.31 | 1.40 | (0.42-4.61) |

| | | | | | |
|---|---|---|---|---|---|
| Dry Clean Products | 3/73 | 8/106 | 0.54 | 0.45 | (0.11-1.80) |
| **Anesthetic Gas** | 0/76 | 4/110 | - | - | |
| **Overall Electro-magnetic Factors** | 25/51 | 33/81 | 1.20 | 1.11 | (0.56-2.21) |
| Electric and Electronic Equipment | 21/55 | 31/83 | 1.02 | 0.94 | (0.47-1.91) |
| Electromagnetic Fields | 10/66 | 12/102 | 1.29 | 1.24 | (0.48-3.21) |

[a]Crude model; [b]Model adjusted by sex, age, and educational attainment; [c]In men only.

**Table S9.** Odds ratio (OR) with 95% confidence interval (CI) of ALS risk according to occupational history and exposure in Southern Italy province (Catania).

| Questionnaire Section | Cases (y/n) | Controls (y/n) | OR[a] | OR[b] | (95% CI) |
|---|---|---|---|---|---|
| **Occupational History** | | | | | |
| **Working Sector** | | | | | |
| Agriculture Working Sector | 1 | 0 | - | - | |
| Manufacturing Working Sector | 7 | 3 | 3.82 | 2.65 | (0.42-16.77) |
| Service Working Sector | 11 | 18 | Ref. | Ref. | - |
| **Occupational Category** | | | | | |
| Armed Forces | 1 | 1 | 0.67 | ne | |
| Managers | 1 | 1 | 0.67 | ne | |
| Professionals/Intellectuals | 1 | 4 | 0.17 | 0.76 | (0.18-3.18) |
| Technicians and Associate Workers | 3 | 2 | Ref. | Ref. | - |
| Clerical Support Workers | 1 | 3 | 0.22 | 0.29 | (0.09-0.94) |
| Services and Sales Workers | 0 | 2 | - | - | |
| Skilled Agricultural Workers | 1 | 0 | - | - | |
| Craft and Related Trades Workers | 4 | 1 | 2.67 | 1.85 | (0.06-61.32) |
| Plant and Machine Operator Workers | 0 | 0 | - | - | |
| Retired or Other Occupation | 1 | 3 | 0.22 | 0.42 | (0.01-20.97) |
| Housewives | 6 | 4 | 1.00 | 25.13 | (0.42-too high) |
| **Previous Agricultural Work** | 2/17 | 0/21 | - | - | |
| No Agricultural Work | 17 | 21 | Ref. | Ref. | |
| Duration of Work 1-10 years | 1 | 0 | - | - | |
| Duration of Work ≥ 10 years | 1 | 0 | - | - | |
| **Work as Welder** | 0/19 | 1/20 | - | - | |
| **Presence of the Photocopier at Work** | 2/17 | 6/15 | 0.29 | 0.15 | (0.01-1.67) |
| **Military Service[c]** | 6/3 | 2/3 | 3.00 | 25.90 | (0.26-too high) |
| **Occupational Exposure to Toxic Agents** | | | | | |
| **Metals/Metalloids** | 6/13 | 3/18 | 2.77 | 3.05 | (0.40-23.12) |
| Lead | 5/14 | 3/18 | 2.14 | 2.41 | (0.32-18.08) |
| Mercury | 2/17 | 1/20 | 2.35 | 1.52 | (0.06-35.75) |
| Selenium | 0/19 | 1/20 | - | - | |
| Cadmium | 0/19 | 0/21 | - | - | |
| **Overall Pesticides** | 2/17 | 4/17 | 0.50 | 0.85 | (0.10-6.87) |
| Insecticides | 2/17 | 3/18 | 0.71 | 1.12 | (0.13-9.81) |
| Herbicides | 1/18 | 2/19 | 0.53 | 0.95 | (0.06-15.25) |
| Fungicides | 1/18 | 0/21 | - | - | |
| **Overall Chemicals/Solvents** | 8/11 | 7/14 | 1.45 | 1.46 | (0.30-6.97) |
| Oil Paints | 3/16 | 3/18 | 1.12 | 0.69 | (0.08-6.24) |
| Thinners | 4/15 | 4/17 | 1.13 | 0.92 | (0.15-5.83) |
| Paint Removers | 3/16 | 2/19 | 1.78 | 0.62 | (0.05-6.96) |
| Paints | 2/17 | 3/18 | 0.71 | 0.13 | (0.91-1.97) |
| Adhesives | 2/17 | 3/18 | 0.71 | 0.56 | (0.06-5.51) |
| Print Inks and Dyes | 3/16 | 4/17 | 0.80 | 0.48 | (0.07-3.24) |
| Lubricating Oils | 1/18 | 3/18 | 0.33 | 0.18 | (0.01-2.83) |
| Refrigerants, Antifreezes, and Cooling Liquids | 1/18 | 2/19 | 0.53 | 0.39 | (0.02-6.74) |
| Degreasing Agents | 1/18 | 3/18 | 0.33 | 0.37 | (0.03-4.65) |

| | | | | | |
|---|---|---|---|---|---|
| Solvents (e.g., toluene, xylene) | 3/16 | 1/20 | 3.75 | 3.65 | (0.25-52.37) |
| Dry Clean Products | 0/19 | 1/20 | - | - | |
| **Anesthetic Gas** | 1(18 | 2/19 | 0.53 | 0.40 | (0.02-7.15) |
| **Overall Electro-magnetic Factors** | 4/15 | 4/17 | 1.13 | 0.39 | (0.05-3.01) |
| Electric and Electronic Equipment | 2/17 | 4/17 | 0.50 | 0.21 | (0.02-1.90) |
| Electromagnetic Fields | 3/16 | 0/21 | - | - | |

[a]Crude model; [b]Model adjusted by sex, age, and educational attainment; [c]In men only.

**Table S10.** Odds ratio (OR) with 95% confidence interval (CI) of ALS risk according residential information and non-occupational use of pesticides in Northern Italy provinces (Modena, Novara and Reggio Emilia).

| Questionnaire Section | Cases (y/n) | Controls (y/n) | OR[a] | OR[b] | (95% CI) |
|---|---|---|---|---|---|
| **Residential Information** | | | | | |
| Ever Lived in the Countryside or Had a Farm | 39/37 | 44/70 | 1.68 | 1.45 | (0.78-2.71) |
| Duration ≥ 10 years | 34/42 | 39/75 | 1.56 | 1.36 | (0.72-2.56) |
| Ever Lived less than 3 km from Water Bodies | 38/38 | 39/75 | 1.92 | 1.94 | (1.05-3.56) |
| Having Lived near Waste Incinerator | 5/70 | 5/109 | 1.87 | 1.87 | (0.54-6.48) |
| Having Lived near Waste Disposal Site | 7/69 | 13/101 | 0.79 | 0.63 | (0.23-1.73) |
| Having Lived near Overhead Power Lines | 18/58 | 14/100 | 2.22 | 2.21 | (1.00-4.88) |
| **Use of Pesticides (not Occupational)** | | | | | |
| Have Pets Living Indoor | 20/56 | 38/76 | 0.71 | 0.65 | (0.33-1.26) |
| Ever had Pets Living Indoor | 59/17 | 87/27 | 1.08 | 1.20 | (0.59-2.44) |
| Use of Flea and Tick Products on Pets | 32/44 | 47/67 | 1.04 | 0.98 | (0.53-1.79) |
| Overall Pesticides Use on Plants | 19/57 | 41/73 | 0.59 | 0.56 | (0.29-1.08) |
| Pesticides Use on Meadow | 11/65 | 22/92 | 0.71 | 0.68 | (0.30-1.53) |
| Pesticides Use on Outdoor Plants | 14/62 | 31/83 | 0.60 | 0.60 | (0.29-1.24) |
| Pesticides Use on Indoor Plants | 3/73 | 6/108 | 0.74 | 0.79 | (0.18-3.45) |
| Overall Pesticides Use on Animals | 32/44 | 60/54 | 0.65 | 0.69 | (0.38-1.25) |
| Pesticides Use on Flying Bugs | 30/46 | 54/60 | 0.72 | 0.76 | (0.41-1.39) |
| Pesticides Use on Ground Bugs | 13/63 | 34/80 | 0.49 | 0.51 | (0.24-1.07) |
| Pesticides Use on Rodents and Rats | 5/71 | 17/97 | 0.40 | 0.41 | (0.14-1.18) |
| Pesticides Use on Other Animals | 15/61 | 23/91 | 0.97 | 0.95 | (0.45-1.99) |
| Bug Disinfection in the Residence | 5/71 | 8/106 | 0.93 | 1.04 | (0.31-3.49) |
| History of Pesticides Use by the Municipality | 30/46 | 44/70 | 1.04 | 0.93 | (0.50-1.72) |

[a]Crude model; [b]Model adjusted by sex, age, and educational attainment.

**Table S11.** Odds ratio (OR) with 95% confidence interval (CI) of ALS risk according residential information and non-occupational use of pesticides in Southern Italy province (Catania).

| Questionnaire Section | Cases (y/n) | Controls (y/n) | OR[a] | OR[b] | (95% CI) |
|---|---|---|---|---|---|
| **Residential Information** | | | | | |
| Ever Lived in the Countryside or Had a Farm | 3/16 | 3/18 | 1.12 | 1.33 | (0.19-9.13) |
| Duration ≥ 10 years | 3/16 | 1/29 | 3.75 | 5.55 | (0.38-80.79) |
| Ever Lived less than 3 km from Water Bodies | 3/16 | 1/20 | 3.75 | 3.05 | (0.24-38.36) |
| Having Lived near Waste Incinerator | 0/19 | 0/21 | - | - | |
| Having Lived near Waste Disposal Site | 0/19 | 2/19 | - | - | |
| Having Lived near Overhead Power Lines | 3/16 | 0/21 | - | - | |
| **Use of Pesticides (not Occupational)** | | | | | |
| Have Pets Living Indoor | 8/11 | 4/17 | 3.09 | 7.09 | (0.94-53.13) |
| Ever had Pets Living Indoor | 13/6 | 16/5 | 0.68 | 0.78 | (0.17-3.68) |
| Use of Flea and Tick Products on Pets | 8/11 | 6/15 | 1.82 | 4.78 | (0.72-31.93) |

| | | | | | |
|---|---|---|---|---|---|
| Overall Pesticides Use on Plants | 2/17 | 2/19 | 1.12 | 0.82 | (0.06-11.61) |
| Pesticides Use on Meadow | 0/19 | 2/19 | - | - | |
| Pesticides Use on Outdoor Plants | 2/17 | 0/21 | - | - | |
| Pesticides Use on Indoor Plants | 0/19 | 0/21 | - | - | |
| Overall Pesticides Use on Animals | 8/11 | 8/13 | 1.18 | 0.67 | (0.10-4.66) |
| Pesticides Use on Flying Bugs | 4/15 | 8/15 | 0.43 | 0.08 | (0.00-1.38) |
| Pesticides Use on Ground Bugs | 7/11 | 4/17 | 3.09 | 2.61 | (0.48-14.15) |
| Pesticides Use on Rodents and Rats | 1/18 | 2/19 | 0.53 | 0.39 | (0.02-7.42) |
| Pesticides Use on Other Animals | 2/17 | 2/19 | 1.12 | 1.94 | (0.18-20.71) |
| Bug Disinfection in the Residence | 1/18 | 0/21 | - | - | |
| History of Pesticides Use by the Municipality | 10/9 | 5/16 | 3.56 | 7.59 | (1.29-44.61) |

[a]Crude model; [b]Model adjusted by sex, age, and educational attainment.

**Table S12.** Odds ratio (OR) with 95% confidence interval (CI) of ALS risk according to occupational information without carriers of *C9orf72* mutation.

| Questionnaire Section | Cases (y/n) | Controls (y/n) | OR[a] | OR[b] | (95% CI) |
|---|---|---|---|---|---|
| **Occupational History** | | | | | |
| **Working Sector** | | | | | |
| Agriculture Working Sector | 8 | 8 | 2.06 | 1.78 | (0.57-5.57) |
| Manufacturing Working Sector | 46 | 55 | 1.72 | 1.49 | (0.79-2.76) |
| Service Working Sector | 35 | 72 | Ref. | Ref. | - |
| **Occupational Category** | | | | | |
| Armed Forces | 2 | 2 | 1.29 | 2.08 | (0.24-18.16) |
| Managers | 2 | 3 | 0.86 | 1.02 | (0.15-7.12) |
| Professionals/Intellectuals | 5 | 10 | 0.65 | 0.98 | (0.23-4.28) |
| Technicians and Associate Workers | 17 | 22 | Ref. | Ref. | - |
| Clerical Support Workers | 4 | 23 | 0.16 | 0.19 | (0.05-0.76) |
| Services and Sales Workers | 12 | 10 | 1.55 | 1.48 | (0.47-4.61) |
| Skilled Agricultural Workers | 8 | 8 | 1.29 | 1.21 | (0.32-4.63) |
| Craft and Related Trades Workers | 25 | 26 | 1.24 | 0.98 | (0.39-2.46) |
| Plant and Machine Operator Workers | 4 | 7 | 0.74 | 0.56 | (0.13-2.35) |
| Retired or Other Occupation | 5 | 18 | 0.36 | 0.30 | (0.08-1.10) |
| Housewives | 6 | 5 | 1.55 | 1.40 | (0.33-6.04) |
| **Previous Agricultural Work** | 20/69 | 13/122 | 2.72 | 2.41 | (1.09-5.36) |
| No Agricultural Work | 69 | 122 | Ref. | Ref. | |
| Duration of Work 1-10 years | 8 | 5 | 2.83 | 2.31 | (0.70-7.59) |
| Duration of Work ≥ 10 years | 12 | 8 | 2.65 | 2.48 | (0.92-6.69) |
| **Work as Welder** | 7/82 | 5/130 | 2.22 | 1.80 | (0.53-6.13) |
| **Presence of the Photocopier at Work** | 21/67 | 44/91 | 0.68 | 0.78 | (0.38-1.60) |
| **Military Service**[c] | 30/17 | 46/25 | 0.96 | 0.88 | (0.39-1.97) |
| **Occupational Exposure to Toxic Agents** | | | | | |
| **Metals/Metalloids** | 23/66 | 13/122 | 3.27 | 4.19 | (1.84-9.55) |
| Lead | 21/68 | 13/122 | 2.90 | 3.62 | (1.58-8.29) |
| Mercury | 5/84 | 2/133 | 3.96 | 4.81 | (0.87-26.53) |
| Selenium | 3/86 | 2/133 | 2.32 | 2.76 | (0.43-17.82) |
| Cadmium | 2/87 | 2/133 | 1.53 | 1.98 | (0.26-15.37) |
| **Overall Pesticides** | 21/68 | 25/110 | 1.36 | 1.33 | (0.68-2.60) |
| Insecticides | 21/68 | 23/112 | 1.50 | 1.47 | (0.74-2.90) |
| Herbicides | 12/77 | 13/122 | 1.46 | 1.50 | (0.64-3.55) |
| Fungicides | 11/78 | 9/126 | 1.97 | 1.91 | (0.73-4.98) |
| **Overall Chemicals/Solvents** | 41/48 | 50/85 | 1.45 | 1.47 | (0.81-2.66) |
| Oil Paints | 7/82 | 9/126 | 1.20 | 1.20 | (0.41-3.54) |
| Thinners | 26/63 | 22/113 | 2.12 | 2.27 | (1.12-4.60) |
| Paint Removers | 16/73 | 15/120 | 1.75 | 1.93 | (0.84-4.43) |

| | | | | | |
|---|---|---|---|---|---|
| Paints | 19/70 | 19/116 | 1.66 | 1.89 | (0.85-4.17) |
| Adhesives | 12/77 | 18/117 | 1.01 | 1.08 | (0.47-2.47) |
| Print Inks and Dyes | 9/80 | 15/120 | 0.90 | 0.95 | (0.38-2.24) |
| Lubricating Oils | 17/72 | 19/116 | 1.44 | 1.53 | (0.69-3.39) |
| Refrigerants, Antifreezes, and Cooling Liquids | 7/82 | 13/122 | 0.80 | 0.86 | (0.31-2.41) |
| Degreasing Agents | 11/78 | 16/119 | 1.05 | 1.07 | (0.46-2.51) |
| Solvents (e.g., toluene, xylene) | 8/81 | 8/127 | 1.57 | 1.71 | (0.58-5.01) |
| Dry Clean Products | 3/86 | 9/126 | 0.49 | 0.44 | (0.11-1.73) |
| **Anesthetic Gas** | 1/88 | 6/129 | 0.24 | 0.32 | (0.04-2.91) |
| **Overall Electro-magnetic Factors** | 29/63 | 37/98 | 1.09 | 1.07 | (0.56-2.03) |
| Electric and Electronic Equipment | 21/68 | 35/100 | 0.88 | 0.85 | (0.44-1.67) |
| Electromagnetic Fields | 12/77 | 12/122 | 1.60 | 1.74 | (0.70-4.30) |

[a]Crude model; [b]Model adjusted by sex, age, and educational attainment; [c]In men only.

**Table S13.** Odds ratio (OR) with 95% confidence interval (CI) of ALS risk according residential information and non-occupational use of pesticides without carriers of *C9orf72* mutation.

| Questionnaire Section | Cases (y/n) | Controls (y/n) | OR[a] | OR[b] | (95% CI) |
|---|---|---|---|---|---|
| **Residential Information** | | | | | |
| Ever Lived in the Countryside or Had a Farm | 41/48 | 47/88 | 1.60 | 1.44 | (0.82-2.54) |
| Duration ≥ 10 years | 36/53 | 40/95 | 1.61 | 1.47 | (0.82-2.64) |
| Ever Lived less than 3 km from Water Bodies | 39/50 | 40/95 | 1.85 | 1.86 | (1.05-3.31) |
| Having Lived near Waste Incinerator | 6/83 | 5/130 | 1.88 | 1.87 | (0.54-6.44) |
| Having Lived near Waste Disposal Site | 7/82 | 15/120 | 0.68 | 0.62 | (0.23-1.65) |
| Having Lived near Overhead Power Lines | 20/69 | 14/121 | 2.51 | 2.45 | (1.14-5.27) |
| **Use of Pesticides (not Occupational)** | | | | | |
| Have Pets Living Indoor | 27/62 | 42/93 | 0.96 | 0.91 | (0.49-1.67) |
| Ever had Pets Living Indoor | 68/21 | 103/32 | 1.01 | 1.15 | (0.60-2.19) |
| Use of Flea and Tick Products on Pets | 39/50 | 53/82 | 1.21 | 1.21 | (0.69-2.12) |
| Overall Pesticides Use on Plants | 21/68 | 43/92 | 0.66 | 0.67 | (0.36-1.24) |
| Pesticides Use on Meadow | 11/78 | 24/111 | 0.65 | 0.65 | (0.30-1.43) |
| Pesticides Use on Outdoor Plants | 16/73 | 31/104 | 0.74 | 0.74 | (0.37-1.49) |
| Pesticides Use on Indoor Plants | 3/86 | 6/129 | 0.75 | 0.79 | (0.18-3.42) |
| Overall Pesticides Use on Animals | 37/52 | 68/67 | 0.70 | 0.81 | (0.46-1.41) |
| Pesticides Use on Flying Bugs | 32/57 | 62/73 | 0.66 | 0.74 | (0.42-1.30) |
| Pesticides Use on Ground Bugs | 19/70 | 38/97 | 0.69 | 0.80 | (0.42-1.54) |
| Pesticides Use on Rodents and Rats | 6/83 | 19/116 | 0.44 | 0.49 | (0.18-1.29) |
| Pesticides Use on other Animals | 16/73 | 25/110 | 0.96 | 0.94 | (0.46-1.91) |
| Bug Disinfection in the Residence | 6/83 | 8/127 | 1.15 | 1.29 | (0.42-4.00) |
| History of Pesticides Use by the Municipality | 38/51 | 49/86 | 1.31 | 1.18 | (0.67-2.08) |

[a]Crude model; [b]Model adjusted by sex, age, and educational attainment.
